# The importance of method selection when estimating diet composition with quantitative fatty acid signature analysis

**DOI:** 10.1371/journal.pone.0308283

**Published:** 2025-01-28

**Authors:** Jeffrey F. Bromaghin, Todd C. Atwood, Karyn D. Rode

**Affiliations:** Alaska Science Center, U.S. Geological Survey, Anchorage, AK, United States of America; Wrocław University of Environmental and Life Sciences: Uniwersytet Przyrodniczy we Wroclawiu, POLAND

## Abstract

Quantitative fatty acid signature analysis (QFASA) is a common method of estimating the composition of prey species in the diets of consumers from polar and temperate ecosystems in which lipids are an important source of energy. A key characteristic of QFASA is that the large number of fatty acids that typically comprise lipids permits the dietary contributions of a correspondingly large number of prey types to be estimated. Several modifications to the original QFASA methods have been suggested in the literature and a significant extension of the original model published in 2017 allows simultaneous estimation of both diet proportions and calibration coefficients, which are metabolic constants in the model whose values must otherwise be estimated in independent feeding experiments. However, comparisons of diet estimates obtained using different estimation options have been limited. QFASA has been used to estimate the diet composition of several polar bear (*Ursus maritimus*) subpopulations, including the Southern Beaufort Sea (SBS) subpopulation. Prior QFASA estimates of SBS polar bear diet composition have most often been obtained using variations of the original QFASA model. We investigated the influence of variations in QFASA analytical methods on diet estimates by re-estimating the diet composition of polar bears from the Alaska portion of the SBS using three different methods and found that differences among the three sets of estimates were substantial. Our results illustrate how important the careful and deliberate selection of QFASA methods can be and we provide some guidance on techniques one might use to evaluate options.

## Introduction

The method of estimating the diet composition of predators (consumers) called “quantitative fatty acid signature analysis” (QFASA) was proposed in 2004 [1]. The method is based on fatty acid “signatures” (FASs), which are compositional vectors of proportions (percentages) representing the relative abundance of fatty acids (FAs), i.e., proportions (percentages) that sum to 1.0 (100%), in lipid tissue [2]. The original QFASA model adjusts predator FASs to account for metabolic processes associated with ingestion, routing and storage of FAs using multiplicative constants called “calibration coefficients” (CCs) [1]. In the terminology of Bromaghin et al. [3], the original model accounts for metabolic processes by using CCs to transform predator signatures from the “predator space” to the “prey space”, inverting the metabolic effects of predator ingestion. The CCs are constants that have generally been estimated in feeding experiments conducted independently from diet estimation e.g., [1, 4, 5] and often applied to non-model species e.g. [1, 6–10]. CCs are conceptually identical to the additive constants called “discrimination factors”, “trophic enrichment factors”, or “diet-tissue discriminant factors” used in stable isotope mixing models, e.g. [11–13]. A predator FAS transformed to the prey space is modeled as a weighted average of mean FASs of potential prey (species or other classes of prey) and diet composition estimates are taken as the weights that minimize a measure of distance between the observed and modeled predator FASs [1].

The original QFASA model used the Kullback-Leibler distance [1], which involves both the absolute and relative difference between two proportions, but the Aitchison distance, which is based only on relative differences, is a commonly-used distance between compositional vectors [14] and Stewart and Field [15] may have been the first to propose its use in QFASA; the chi-square distance [16] has been used less frequently and is not considered here. QFASA estimates based on the Aitchison distance tend to have lower bias and variance and be insensitive to the estimation space (predator or prey) used than estimates based on the Kullback-Leibler distance [3], and also to be more robust to bias caused by imperfect knowledge of the CCs [17].

FASs are inherently compositional and the sum of all FA proportions is therefore 1 (100 for percentages). However, it is common for the FAs used in QFASA to be restricted to those that are strongly linked to diet (the so-called dietary or extended dietary FAs) and FAs that are found to misallocate among key prey types are commonly excluded, e.g., [1, 18, 19]. In such cases, the traditional analytical approach has been to normalize (re-scale) the subset of proportions selected for diet modeling so that they sum to 1 using the multiplicative method [1, 20]. However, Bromaghin et al. [21] found that normalizing FASs introduces bias and instead recommended that FASs be “augmented” with an additional FA proportion equal to the sum of all the FA proportions from the complete vector to be excluded from the model, so that FAS values between individuals are not distorted and FASs remain compositional.

In addition to the above analytical options for the original QFASA method, an important extension of the original model allows the CCs to be estimated simultaneously with the diet proportions [22]. This extension is a significant methodological advancement because CCs must otherwise be estimated in independent feeding experiments, which require substantial infrastructure and are time-consuming to complete and consequently have only been completed for a few species. Even if a feeding experiment(s) has been completed with the target species, many aspects of the experimental design such as the selection of food items and the growth stage or activity level of captive animals might lead to estimates of CCs that are not accurate for free-ranging predator populations. The extended model [22] therefore eliminates what is arguably the primary limitation of the original QFASA model.

QFASA has been used to estimate the diet composition of polar bears (*Ursus maritimus*) from subpopulations throughout the Arctic, e.g. [5, 19, 23–28], as well as for the Southern Beaufort Sea (SBS) subpopulation [29–31] whose range includes the northern coast of Alaska [32]. The large number of fatty acids in lipid tissue allows QFASA to estimate numerous prey species in the diets of individual animals. DNA methods applied to fecal samples also have the potential to estimate contributions from numerous prey species e.g., [33–35], while stable isotope diet models are often underdetermined because of the smaller number of biomarkers that are typically used [36]. Diet estimates for individual predators are particularly useful in understanding the effects of environmental conditions on diet and the role that diet plays in affecting body condition and health, e.g., [37]. Most polar bear studies to date have used the methods of the original QFASA model [1], although the Aitchison distance measure has been used with the original QFASA model in a few studies [19, 28, 31].

We investigate the degree to which the selection of a QFASA model and the various estimation options mentioned above can lead to differences in diet composition estimates using polar bears from Alaska’s SBS subpopulation as an example predator. We estimate diet composition for polar bears using three different combinations of estimation options [38]. The diet compositions of these bears were previously estimated using the original QFASA model [29, 30]. We also describe diagnostic methods that investigators may find helpful in evaluating estimation options.

## Methods

Fat biopsy samples were collected from 505 polar bears captured or biopsy darted [39] from the Alaska portion of the SBS subpopulation range [32] during March, April, or May from 2004 to 2016 [40]. Following [29], we based our estimates on 31 fatty acids that are primarily acquired from the diet (termed dietary fatty acids [1]) and therefore thought to be informative with respect to diet [1, 18]: 16:2n-6, 16:2n-4, 16:3n-6, 16:3n-4, 16:4w3, 16:4n-1, 18:2n-6, 18:2n-4, 18:3n-6, 18:3n-4, 18:3n-3, 18:3n-1, 18:4n-3, 18:4n-1, 20:1n-11, 20:1n-9, 20:1n-7, 20:2n-6, 20:3n-6, 20:4n-6, 20:3n-3, 20:4n-3, 20:5n-3, 22:1n-11, 22:1n-9, 22:1n-7, 21:5n-3, 22:4n-6, 22:5n-6, 22:4n-3, and 22:6n-3.

Fatty acid data from four potential prey species, ringed seal (*Pusa hispida*), bearded seal (*Erignathus barbatus*), bowhead whale (*Balaena mysticetus*), and beluga whale (*Delphinapterus leucas*), were compiled from three online sources [41–43] and one unpublished source [24]. Data from additional species thought to not contribute meaningfully to diet during winter and spring were excluded (ribbon seal *Histriophoca fasciata*, spotted seal *Phoca largha*, and Pacific walrus *Odobenus rosmarus*) [37 Appendix S2]. For ringed seals, there were 9 subadult, 5 pup, and 1 unknown-age Cooper records [41, 44], 19 non-pup and 5 pup US Geological Survey (USGS) records [43], 8 US Fish and Wildlife Service (USFWS) records without age-class information [42], and 89 Thiemann records without age-class information [23]. For bearded seals, there were 7 adult, 12 subadult, and 6 unknown-age Budge records [41, 44], 6 adult, 23 subadult, and 1 pup Cooper records [41, 44], 13 non-pup USGS records [43], 28 USFWS records without age-class information [42], and 20 Thiemann records without age-class information [23]. There were 64 bowhead whale records [41, 45] and 29 beluga whale records. The Budge, Cooper, USFWS, and USGS records were collected in the Chukchi Sea and the Thiemann records were collected in the Beaufort Sea.

We used R version 4.3.2 [46] and the “adonis2” function of the vegan package [47] to conduct permutation analysis of variance (ANOVA) tests of the equality of various subsets of the ringed and bearded seal FAS data. For these tests, FASs consisted of the proportions of the 31 dietary fatty acids mentioned above and an augmented proportion equal to 1 minus the sum of the 31 proportions so that all 32 proportions summed to 1 [21]. The tests were based on the Aitchison distance measure and the number of replicates used in each test was approximately equal to the number of unique permutations of the data among potential prey groups, with a maximum of 100,000 replicates to limit computation time. The results of these tests were used to guide formation of final prey groups for purposes of diet estimation. Our use of age class information to potentially stratify seal prey was largely motivated by Rode et al. [37], who used pup and nonpup prey groups for ringed and bearded seals. We note that the DIMAC clustering algorithm [48] would allow one to investigate the existence of clusters of similar signatures within prey data not necessarily associated with recognized categorical covariates such as sex and age classes, but that more general approach was not of interest in this analysis.

Both polar bear and prey FASs were prepared for analysis by replacing proportions equal to zero with a value equal to 90% of the smallest non-zero proportion in the prey FAS data. Any FASs that had values of zero replaced with a small constant were then normalized (re-scaled) using the multiplicative method [20] so that all proportions summed to 1. All FASs, including those with no zeros replaced, were then either normalized or augmented, as previously described, depending on which of the three sets of analysis options (Table 1) was being implemented. The power of QFASA to differentiate between prey groups was assessed using leave-one-prey-out (LOPO) analyses [21], a cross-validation algorithm that measures the proportion of prey FASs that are attributed to the correct prey group. For a given set of prey groups, the proportion of polar bear FAS proportions smaller than the minimum or larger than the maximum mean prey group proportion was computed as an indicator of potential assumption violations [3, 22, 49]. We also computed the proportion of the distance between all pairs of prey FASs associated with pairs of prey from a common prey group; a statistic ψ that has not previously been used,

$$\psi =\frac{\sum_{i=1}^{G}\sum_{y=1}^{n_{i}-1}\sum_{y^{*}=y+1}^{n_{i}}D\left(FAS_{y},FAS_{y^{*}}\right)}{{\left(\sum_{i=1}^{G}\sum_{y=1}^{n_{i}-1}\sum_{y^{*}=y+1}^{n_{i}}D\left(FAS_{y},FAS_{y^{*}}\right)+\sum_{i=1}^{G-1}\sum_{i^{*}=i+1}^{G}\sum_{y=1}^{n_{i}}\sum_{y^{*}=1}^{n_{i^{*}}}D\left(FAS_{y},FAS_{y^{*}}\right)\right)}^{\prime }}$$

where G is the number of prey groups, n_k_ is the sample size of prey group k, and D is a distance between two FASs. Note that the numerator of ψ is the sum of the within group distances and the denominator is the sum of the within and between group distances. Intuitively, one would expect estimation accuracy to be high when ψ is small, i.e., when FASs are similar within prey groups and highly differentiated between prey groups [49].

**Table 1:** Combinations of quantitative fatty acid signature analysis (QFASA) estimation options (Methods) used to estimate the diet compositions of 505 southern Beaufort Sea polar bears (*Ursus maritimus*) sampled during spring (March to May) from 2004 to 2016. Normalized signatures are multiplicatively scaled so that they sum to 1 and augmented signatures have a proportion added so that all sum to 1.

Estimation	Method		
Component	1	2	3
Model	Iverson	Iverson	Bromaghin
Prey groups	4	10	10
Estimation space	prey	predator	predator
Distance measure	Kullback-Leibler	Both, Aitchison selected	Aitchison
Number fatty acids	31	30	31
Signature completion	Normalization	Augmentation	Augmentation
Software	R package qfasar	R package qfasar	R package Solnp

We estimated diet composition using three combinations of model and estimation options (Table 1). For Method 1, we used options as similar as possible to [29]: the original QFASA model [1], the Kullback-Leibler distance, the 31 dietary FAs listed above, FASs were normalized, and estimation was performed in the prey space. To be consistent with [29], prey data were limited to four Beaufort Sea prey groups: the ringed seal and bearded seal records of the Thiemann source [24] and the Budge source [41, 44], the Budge bowhead whale records [41, 46], and the beluga whale records of Thiemann and Budge [41, 44]. The herring and seal-oil CCs of [24] were used. Diet estimation was performed using the qfasar R package [50]. Method 2 options were the same as Method 1 options, except that some options were not selected a priori but rather were selected based on diagnostic results obtained during the course of the analysis. In particular, we compared diagnostic statistics (LOPO, the proportion of predator FAS proportions outside the prey range, and ψ) derived in the prey space with the Kullback-Leibler distance and in the predator space with the Aitchison distance, using signature augmentation in both cases, to help guide selection of a distance measure. The results of permutation ANOVA tests guided formation of prey groups using both Beaufort Sea and Chukchi Sea prey. Any diet estimates to the scale of prey groups were subsequently pooled to species. In addition, we excluded the fatty acid 20:1n-11 because [22] found a data-based estimate of its CC to differ markedly from the value derived from a mink feeding experiment [24], which suggests that conditioning on the feeding-trial CC for this FA might bias estimates. Method 3 used the same prey groups as Method 2 and the 31 dietary FASs used in Method 1, but applied the Bromaghin QFASA model [22] to simultaneously estimate diet compositions and CCs (because the CCs were estimated, FA 20:1n-11 was retained in the FASs). Estimates were made in the predator space using signature augmentation and the Aitchison distance. The Bromaghin model has not yet been implemented in the R package qfasar [50], so the objective function of [22] was minimized using the “solnp” function of the R package Rsolnp [50]. We did not compute the LOPO diagnostic statistic with Method 3 because the CCs would not have been held constant through each iteration of the algorithm, although estimating the CCs using Method 3 and then using the resulting estimates in qfasar to perform a LOPO analysis might be informative.

## Results

The permutation ANOVA results led to the formation of three ringed seal prey groups and five bearded seal prey groups (Table 2). For ringed seals, the pups and subadults of Cooper were not significantly different (Test 1), but the pups and non-pups of USGS were different (Test 2). The pups of Cooper and USGS were not different (Test 3), nor were Cooper’s subadults and the USGS non-pups (Test 4). Consequently, we formed Cooper-USGS (C-G) pup and non-pup groups. A comparison of those two groups with the USFWS and Thiemann sources was significant (Test 5). All pairwise tests among those four sources (Tests 5a-5e) were significant except for the C-R non-pups versus USFWS, so we decided to pool those two sources into one group, resulting in three groups for ringed seals: C-G pups, C-G non-pups & USFWS, and Thiemann. For bearded seals, the FASs of age classes within source were not significantly different (Tests 6–7), but all five sources were significantly different (Tests 8-8j), so we used all five sources as prey groups for diet estimation with Methods 2 and 3. No age or sex class information was available for either bowhead or beluga whales, so the data for these two species were included as two additional prey groups.

**Table 2:** Results of permutation analysis of variance tests of the equality of the fatty acid signatures of various groups of ringed and bearded seals using the Aitchison distance. Signatures consisted of proportions of 31 dietary fatty acids (listed in Methods) and were augmented with an additional proportion equal to 1 minus the sum of the 31 proportions so that all 32 proportions summed to 1 [14]. The number of replicates is approximately equal to the number of unique permutations of the data used in each test, with a maximum of 100,000 to limit computation time. The name associated with each data source is the surname of the lead author of the paper that initially presented the data, except USFWS denotes the U.S. Fish and Wildlife Service (see Methods). TS denotes the test statistic and p-value denotes test significance.

Test	Description	Replicates	TS	p-value
	**Ringed seals**			
1	Cooper: pup (5) x subadult (9)	30,000	2.0168	0.1105
2	USGS: pup (5) x non-pup (19)	43,000	2.8236	0.0302
3	Pups: Cooper x USGS	250	1.7261	0.1355
4	Cooper subadult x USGS non-pup	100,000	1.3415	0.2221
5	C-G non-pup (28) x C-G pup (10) x USFWS (8) x Thiemann (89)	100,000	9.6318	0.0000
5a	C-G non-pup x USFWS	100,000	1.5861	0.1564
5b	C-G non-pup x Thiemann	100,000	11.1616	0.0000
5c	C-G pup x USFWS	44,000	2.6956	0.0157
5d	C-G pup x Thiemann	100,000	20.1459	0.0000
5e	USFWS x Thiemann	100,000	8.8668	0.0002
	**Bearded seals**			
6	Budge: adult (7) x subadult (12)	50,000	1.3571	0.2329
7	Cooper: adult (6) x subadult (23) x pup (1)	100,000	0.6798	0.5175
8	Budge (25) x Cooper (30) x USGS (13) x Thiemann (20) x USFWS (28)	100,000	7.1200	0.0000
8a	Budge x Cooper	100,000	4.9460	0.0002
8b	Budge x USGS	100,000	5.8221	0.0000
8c	Budge x Thiemann	100,000	8.3668	0.0000
8d	Budge x USFWS	100,000	5.7267	0.0001
8e	Cooper x USGS	100,000	4.3963	0.0000
8f	Cooper x Thiemann	100,000	8.5048	0.0000
8g	Cooper x USFWS	100,000	10.4863	0.0000
8h	USGS x Thiemann	100,000	4.9394	0.0005
8i	USGS x USFWS	100,000	6.1491	0.0000
8j	Thiemann x USFWS	100,000	11.4228	0.0000
	**Ringed and bearded seals**			
9	Ringed (135) x bearded (116)	100,000	102.4424	0.0000
9a	The groups of Tests 5 and 8 within species strata	100,000	22.4001	0.0000

With Method 1, the correct LOPO attributions to prey type were ringed seal 0.789, bearded seal 0.806, bowhead whale 0.880, and beluga whale 0.917, with a total across prey groups of 3.392. Over 40% (40.7%) of the predator FAS proportions were outside the range of the mean prey FAS proportions, a sign of assumption violations [3, 22, 50], and ψ = 11.0% of the total distance between all FAS pairs occurred within prey types.

**Fig 1:**
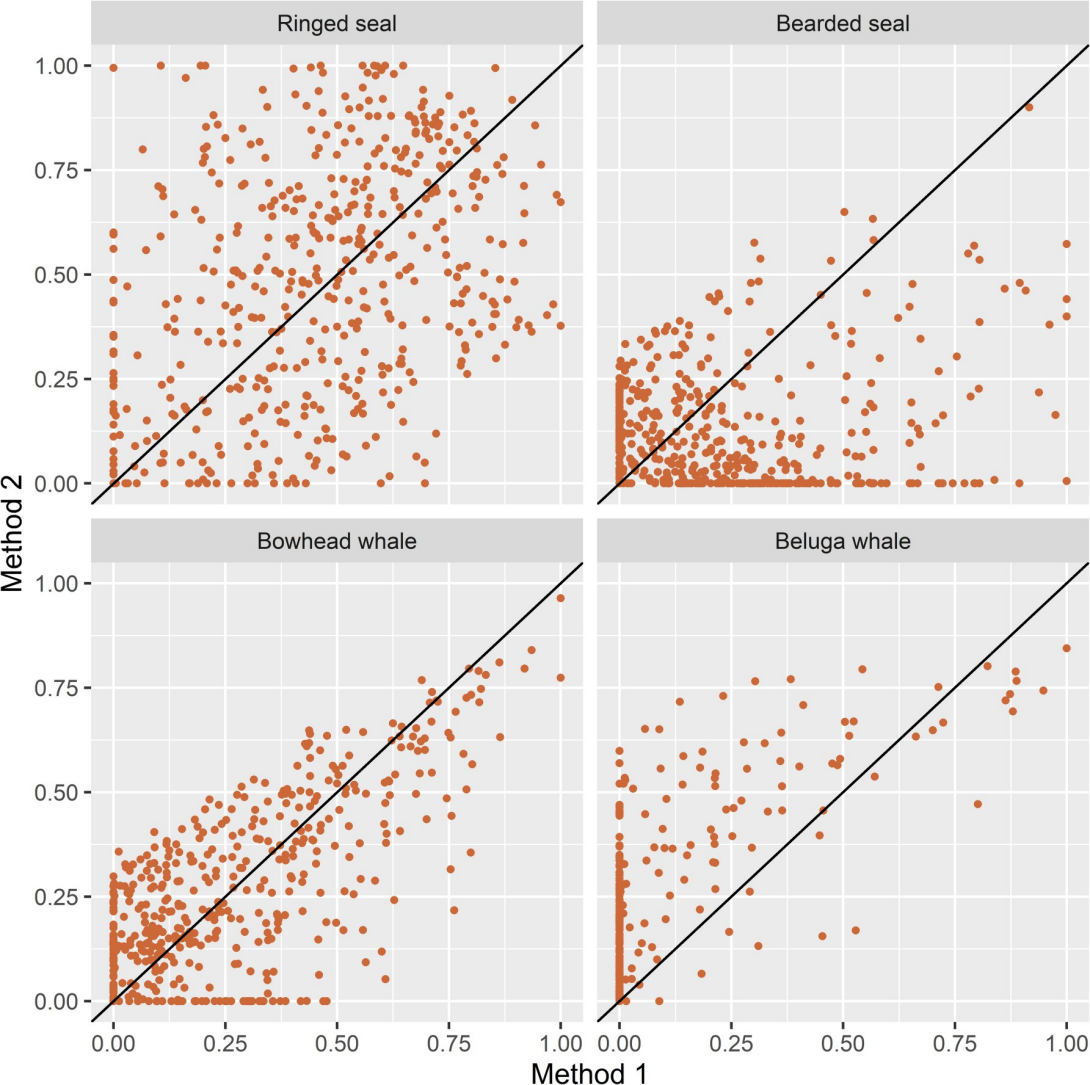
Scatterplots of diet estimates by species obtained with Method 1 and Method 2. Please refer to Table 1 for a summary of estimation options for each method.

With Method 2 and the Kullback-Leibler distance in the prey space, the correct LOPO attributions to prey type were ringed seal 0.851, bearded seal 0.854, bowhead whale 0.879, and beluga whale 0.918, with a total of across species of 3.502. Nearly one third (31.0%) of the predator FAS proportions were outside the range of the mean prey FAS proportions and ψ = 4.0% of the total distance between all FAS pairs occurred within prey types. With the Aitchison distance in the predator space, the correct LOPO attributions to prey were ringed seal 0.844, bearded seal 0.877, bowhead whale 0.974, and beluga whale 0.890, with a total of across species of 3.585. Almost a quarter (24.4%) of the predator FAS proportions were outside the range of the mean prey FAS proportions and ψ = 7.9% of the total distance between all FAS pairs occurred within prey types. The slightly better LOPO results and smaller proportion of polar bear FAS proportions beyond the range of the mean prey FAS proportions led us to select the Aitchison distance measure in the predator space for Method 2. Consequently, Method 2 implements the general recommendations of [3, 17, 21] regarding modifications of the original Iverson QFASA model [1]. The diet estimates obtained using Method 1 and Method 2 are plotted in Fig 1.

**Fig 2:**
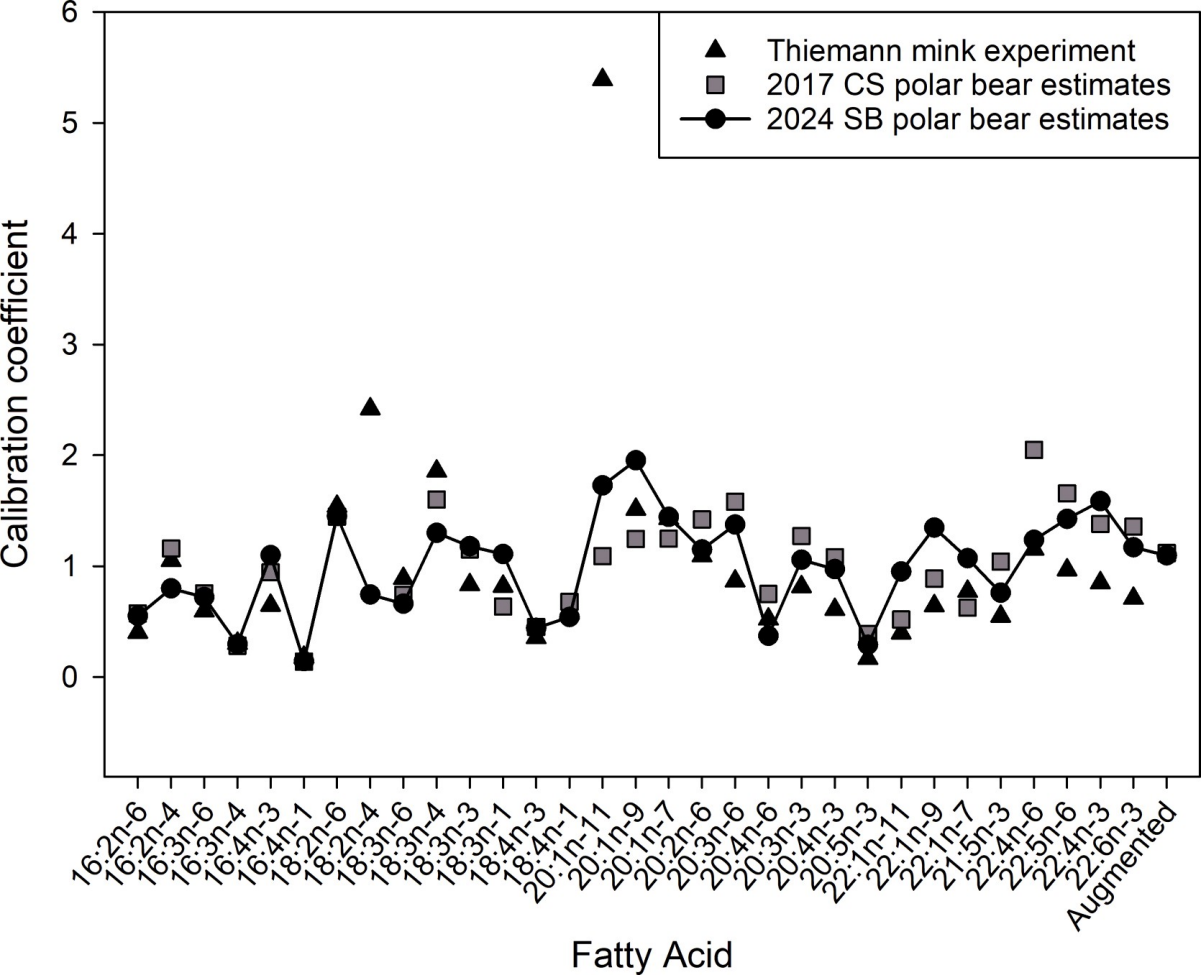
Estimated values of the calibration coefficients for the 31 dietary fatty acids and the augmented proportion used in this study. The Thiemann mink estimates are from [17], the 2017 CS estimates are from [15], and the 2024 SB estimates are from this study.

**Fig 3:**
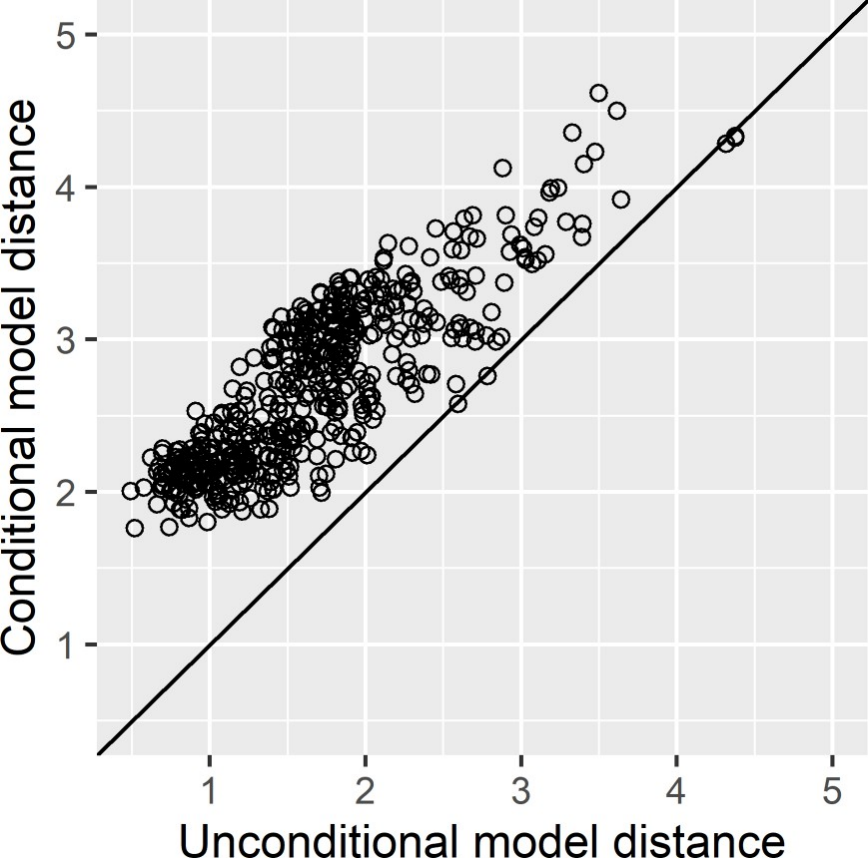
The Aitchison distance between observed and modeled polar bear fatty acid signatures based on Method 2 (conditioned on specific values for calibration coefficients) and Method 3 (calibration coefficients estimated). 500 of 505 individual polar bears had a smaller distance with Method 3. Please refer to Table 1 for a summary of estimation options for each method.

**Fig 4:**
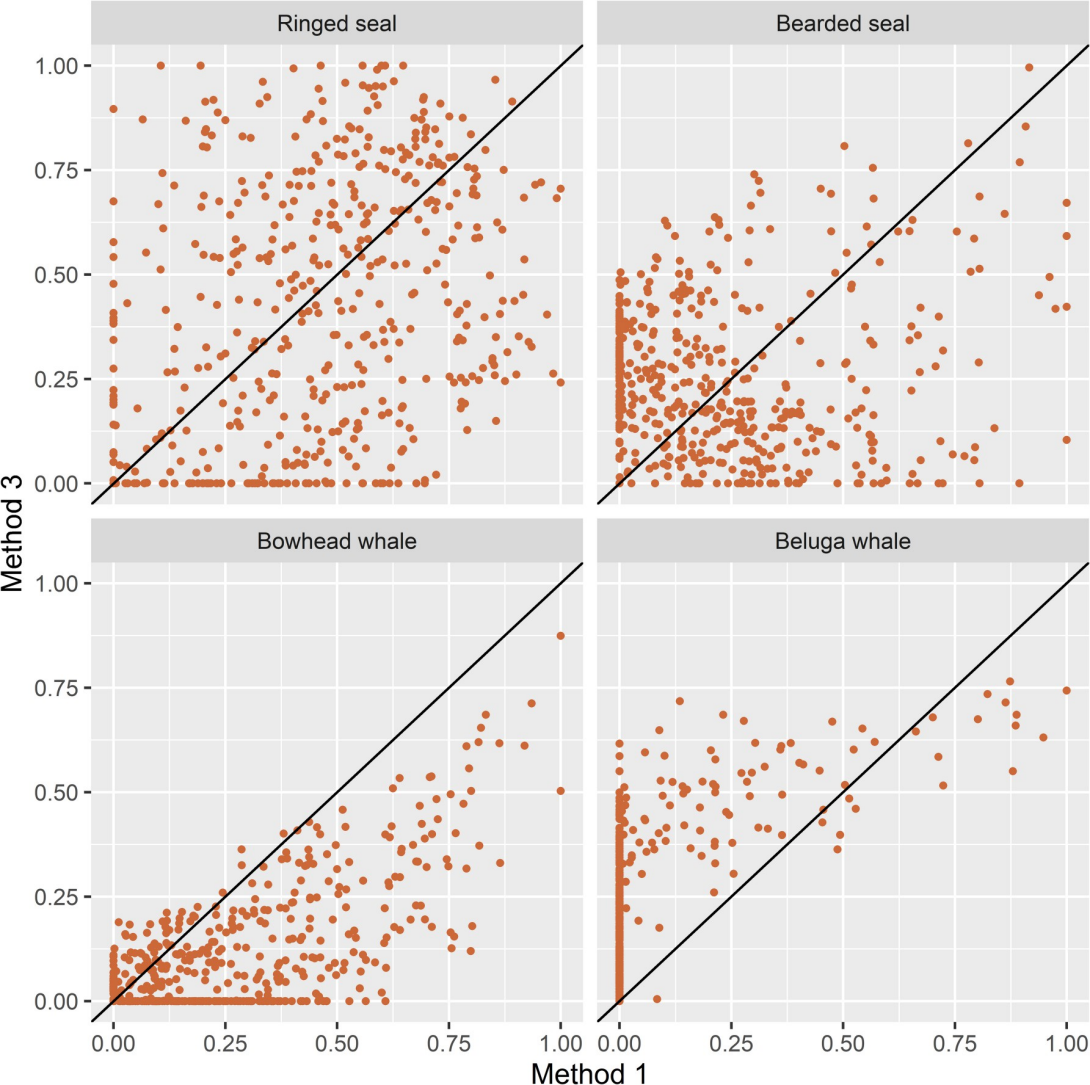
Scatterplots of diet estimates by species obtained with Method 1 and Method 3. Please refer to Table 1 for a summary of estimation options for each method.

**Fig 5:**
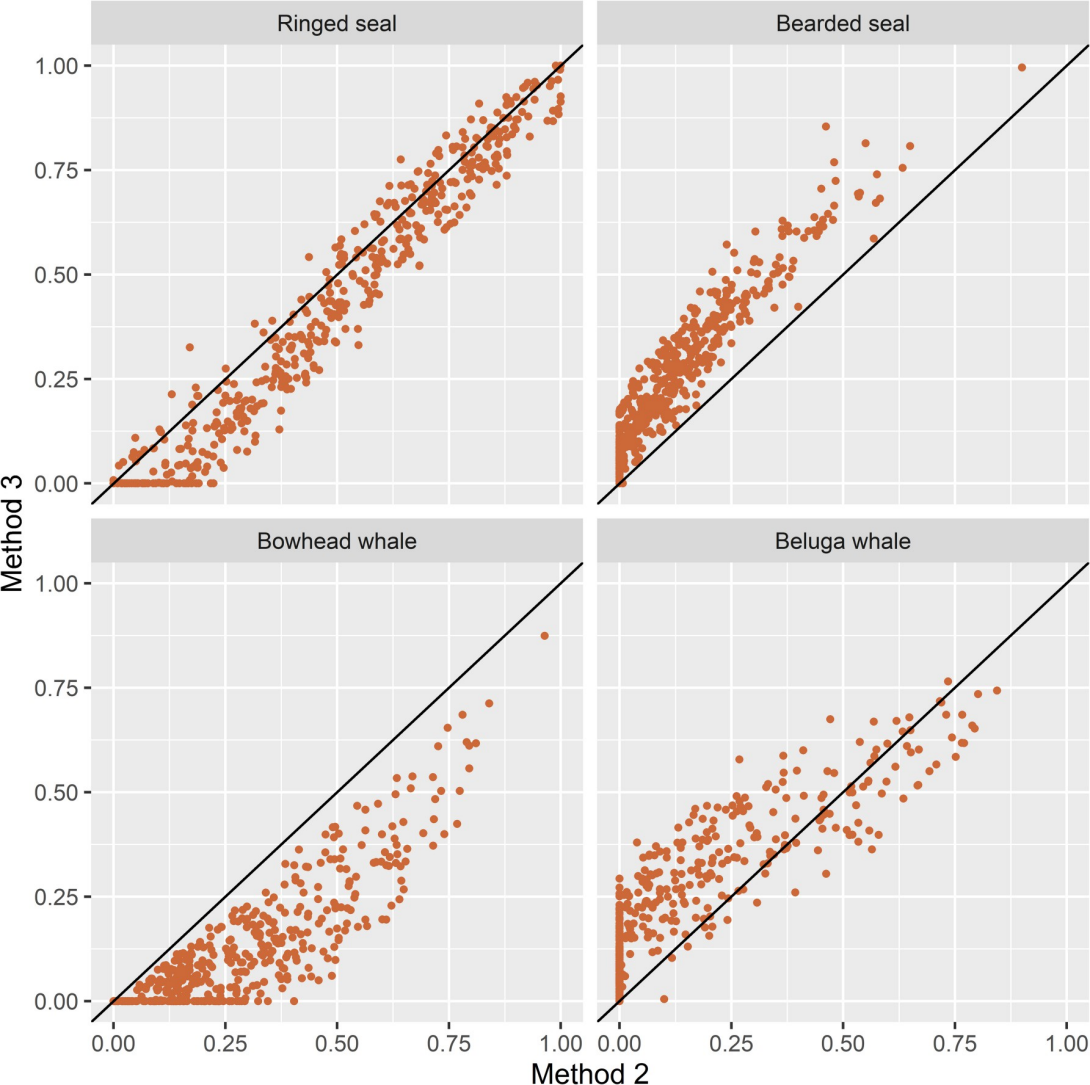
Scatterplots of diet estimates by species obtained with Method 2 and Method 3. Please refer to Table 1 for a summary of estimation options for each method.

Using Method 3, the estimated CCs are similar to estimates previously obtained with Chukchi Sea polar bear FAS data [22] and to estimates from a mink feeding trial [24], though there are large differences among the estimates for a few individual FAs (Fig 2). The proportion of predator FAS proportions outside the range of the mean prey FAS proportions was 15.7%, substantially lower than the values obtained with the other methods. Because Methods 2 and 3 both used the Aitchison distance, we compared how closely these two methods were able to approximate the observed polar bear FASs. Method 3 decreased the Aitchison distance between observed and modeled FASs, summed across bears, by 39% compared to Method 2, with 500 of 505 bears (99%) having a smaller distance under Method 3 (Fig 3), evidence that Method 3 provided a superior fit to the observed polar bear FASs. Diet estimates obtained using Methods 1 and 3 are plotted in Fig 4 and the estimates obtained using Methods 2 and 3 are plotted in Fig 5.

**Fig 6:**
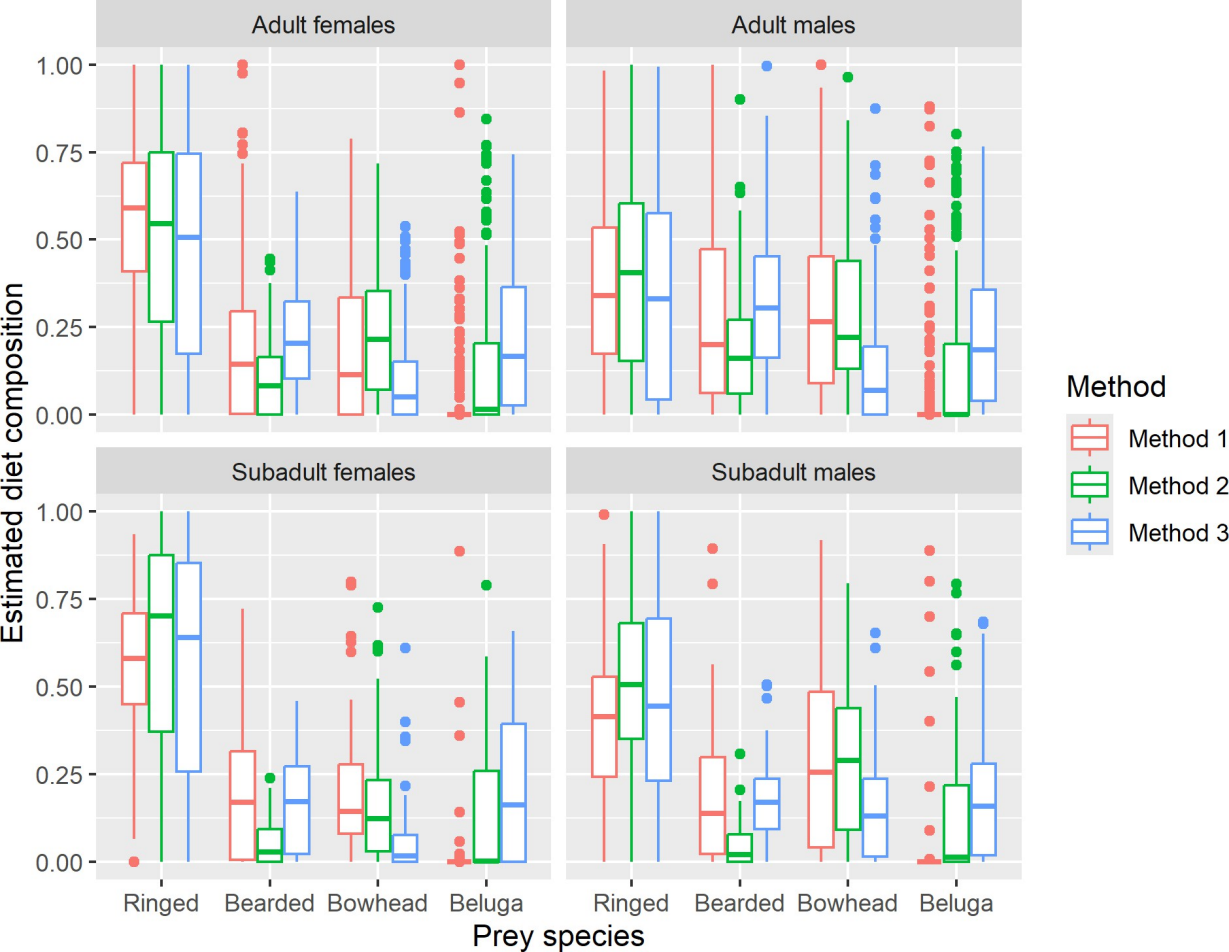
Boxplots of diet composition estimates by method, prey species, and polar bear sex-age class. The line in the middle of each “box” is the median (50th percentile), the lower and upper extents of each box are the 25th and 75th percentiles, and the “whiskers” extend to the most extreme values that are no more than 1.5 times the box width below or above the 25th and 75th percentiles, respectively, with any more extreme values plotted as individual symbols.

**Fig 7:**
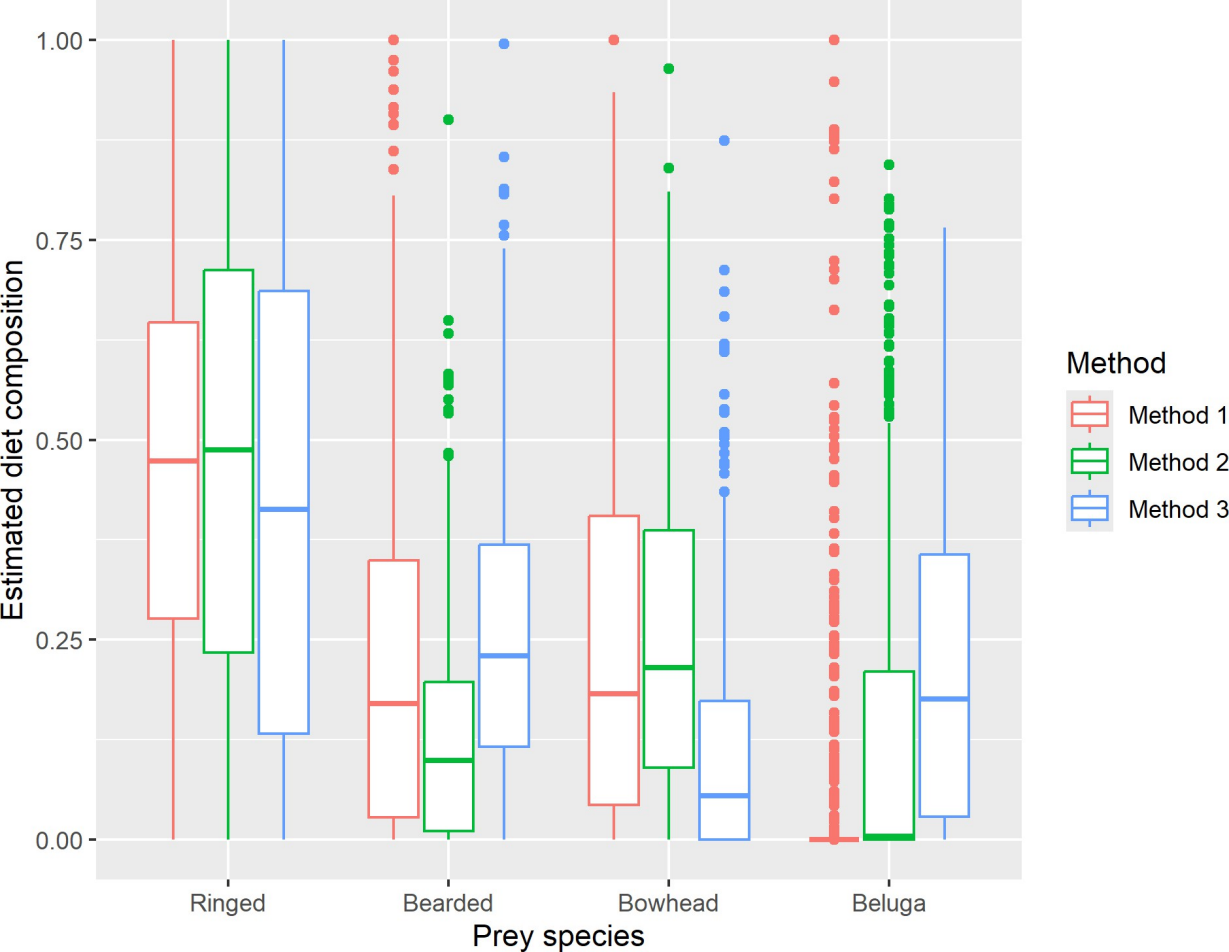
Boxplots of diet composition estimates by method and prey species. The line in the middle of each “box” is the median (50th percentile), the lower and upper extents of each box are the 25th and 75th percentiles, and the “whiskers” extend to the most extreme values that are no more than 1.5 times the box width below or above the 25th and 75th percentiles, respectively, with any more extreme values plotted as individual symbols.

Pearson correlation coefficients were computed between all possible pairs of estimates obtained using the three methods, by prey species (Table 3). In addition to comparing the estimates for individual polar bears in Figs 1, 4 and 5, we constructed boxplots of the estimates to compare differences in the distributions and means of the estimates by adult female, adult male, subadult female, and subadult male age-sex classes (Fig 6) and pooled across age and sex class (Fig 7).

**Table 3:** Pearson correlation coefficients between the diet estimates obtained with each method, by prey species. Please refer to Table 1 for a summary of the estimation options associated with each method.

	Ringed seal	Bearded seal	Bowhead Whale	Beluga whale
Methods 1 & 2	0.3987	0.1468	0.7387	0.7291
Methods 1 & 3	0.3264	0.0699	0.7155	0.6252
Methods 2 & 3	0.9758	0.9407	0.8671	0.8787

## Discussion

The three Methods of diet estimation that we tested, Method 1 which represents the original QFASA model [1], Method 2 which incorporates recommended alternatives to the original model [3, 17, 21], and Method 3 which is based on a generalized model that also allows simultaneous estimation of diet compositions and CCs [22], produced very different estimates of diet composition for Alaska SBS polar bears. In general, Method 2 estimates tended to have larger contributions from beluga whale and somewhat smaller contributions from bearded seal compared to Method 1 (Fig 1). The same patterns are apparent in the distributions of the estimates (Figs 6 and 7). The estimated contributions of the two primary prey species consumed by polar bears in this region, ringed seal and bearded seal, were poorly correlated between Method 1 and the other two methods (r < 0.4 and r < 0.2, respectively, Table 3). In contrast, estimated contributions of all prey species obtained with Methods 2 and 3 were highly correlated (r > 0.85) and their differences were smaller (Table 3). Method 3 estimated larger contributions of bearded seal and beluga whale and smaller contributions of bowhead whale and ringed seal to a lesser extent than Method 2 (Figs 5–7). Method 3 estimates for ringed seal appeared to depend on how prevalent ringed seal was in the diet, with estimates being smaller than Method 2 estimates for bears whose ringed seal estimates were less than approximately 0.5 (Fig 5).

The large differences in polar bear diet estimates we observed highlights the importance of method selection in QFASA applications. We are not aware of any widely accepted guidelines for method selection, but the diagnostic procedures that we performed here as examples should be informative. FA selection is an important aspect of an analysis for which little guidance is available, other than to use FAs that are primarily derived from diet [e.g., 1, 18]. We used diagnostic procedures based on the prey data to help guide the selection of a distance measure and the large differences we observed between Method 1 and Method 2 estimates reveals how important the choice of a distance measure can be. Although the LOPO algorithm [21] is a form of cross-validation that one might reasonably expect to be an informative measure, we have not found LOPO results to be highly predictive of model performance for diet estimation with predator data (unpublished simulation results). The proportion of predator FAS proportions that are outside the range of the mean prey proportions is an important statistic because values larger than 0 provide a clear indication of a violation of QFASA model assumptions [3, 22, 49]; if a predator FAS is to be well approximated by a mixture of prey FASs, its proportions must be within the range of CC-adjusted prey proportions. We introduce the proportion of the summed distance between all pairs of prey FASs that are associated with FAS pairs from a common prey group (ψ) as a potential diagnostic statistic to help select a distance measure or potentially measure the distinctiveness of prey groups within a prey library. Conceptually, the smaller this proportion is, the better the model should be able to distinguish between the identified prey groups, but we have not yet developed guidance for how to interpret specific values. In the absence of compelling evidence to use the Kullback-Leibler distance, the results of [17] suggest use of the Aitchison distance. In the case where competitive options share a common distance measure (as Method 2 and Method 3 in our example), computing the distance between observed and modeled predator FASs provides important information about how well the predator FASs are being approximated (Fig 3). The results of these sorts of diagnostic procedures can help guide the selection of QFASA estimation options, as well as evaluate and document model performance with a particular data set. Simulation results [e.g., 3, 17, 21] can also provide important information about how different estimation options tend to perform. In addition, we note that signature augmentation is generally preferable to signature normalization [21].

The large differences between CC estimates for some individual FAs illustrates how critical the assumption that CC values are known is to the original QFASA model. There is no way to be confident that CCs derived from a feeding experiment are applicable to free-ranging predators. The CCs are used to model predator FASs, so any errors in their values can be expected to bias diet estimates. For this reason, we suspect that the CC-unconditional model [22] should generally be preferred to the original model [1], unless one somehow has strong reasons to believe available CC values are accurate. Although the selection of the QFASA model is obviously an important decision, our results also reveal that the selection of estimation options available for each model, such as signature completion, distance measure, and estimation space, are important determinants of model performance.
